# Shortened leukocyte telomere length in type 2 diabetes mellitus: genetic polymorphisms in mitochondrial uncoupling proteins and telomeric pathways

**DOI:** 10.1186/s40169-016-0089-2

**Published:** 2016-03-07

**Authors:** Yuling Zhou, Zhixin Ning, Yvonne Lee, Brett D. Hambly, Craig S. McLachlan

**Affiliations:** Faculty of Medicine, Rural Clinical School, University of New South Wales, Samuels Building, Level 3, Room 327, Sydney, 2052 Australia; Discipline of Pathology, Bosch Institute, Sydney Medical School, University of Sydney, Sydney, Australia

**Keywords:** Telomere length, Type 2 diabetes, Genetics, Uncoupling protein polymorphisms, Telomere-associated pathway genes

## Abstract

Current debate in type 2 diabetes (T2DM) has focused on shortened leukocyte telomere length (LTL) as the result of a number of possible causes, including polymorphisms in mitochondrial uncoupling proteins (UCPs) leading to oxidative stress, telomere regulatory pathway gene polymorphisms, or as a direct result of associated cardiovascular complications inducing tissue organ inflammation and oxidative stress. There is evidence that a heritable shorter telomere trait is a risk factor for development of T2DM. This review discusses the contribution and balance of genetic regulation of UCPs and telomere pathways in the context of T2DM. We discuss genotypes that are well known to influence the shortening of LTL, in particular OBFC1 and telomerase genotypes such as TERC. Interestingly, the interaction between short telomeres and T2DM risk appears to involve mitochondrial dysfunction as an intermediate process. A hypothesis is presented that genetic heterogeneity within UCPs may directly affect oxidative stress that feeds back to influence the fine balance of telomere regulation, cell cycle regulation and diabetes risk and/or metabolic disease progression.

Telomere length varies from 4–20 kb in humans and telomere shortening is thought to be key mechanistic event in cellular aging [1–3]. Shorter telomeres have been described as a risk factor for many age related chronic conditions [4], including T2DM [5–7]. A current debate concerns whether shorter telomeres are a heritable trait [8], or a consequence of an associated disease process [9, 10] or, in fact due to an age related dysfunction in telomere regulation [11, 12]. Changes in telomere length associated with type 2 diabetes (T2DM) and interactions between mitochondrial function and telomeres represent possible translational targets to treat T2DM by restoring telomere length [13–16].

## Leukocyte telomere length (LTL) is associated with metabolic disease and cardiovascular risk factors

There is certainly a growing trend to report telomere length in population studies that encompass traditional metabolic and cardiovascular risk factors. The large community population Framingham heart study has demonstrated that cardiovascular risk factors such as hypertension and/or insulin resistance are associated with shorter human LTLs [17]. Shortened LTLs have been observed in patients with metabolic syndrome with impaired glycaemic control and increased waist-to-hip ratio [18]. Impaired glycaemic control in both clinical and translational studies, have been associated with increased cellular oxidative stress [19].

## Oxidative stress and LTL in metabolic disease

Diabetes complicated by cardiovascular disease is associated with increased mortality and higher levels of oxidative stress and inflammation, which may accelerate telomere shortening and associated premature cell senescence [20–22]. Importantly, mitochondrial oxidative stress appears to be required to maintain cellular senescence [23]. Additionally, in cancer biology short telomere dysfunction has been associated with a tissue decrease in mitochondrial DNA copy number and, importantly, a decrease in mitochondrial bio-energetic function [24], emphasizing a close inter-relationship between oxidative stress, mitochondrial bio-energetic coupling and telomere length [25–27]. On the other hand, such associations have not been well explored in diabetes. These biological factors influencing regulatory telomere pathways would be amenable to genetic analysis, particularly in populations at risk, such as those that have developed T2DM, with and without cardiovascular risk factors.

## Do short telomeres cause and/or influence progress of T2DM metabolic syndrome?

It is generally accepted that shortened telomeres may independently contribute to clinical metabolic syndrome progression via directly affecting cellular metabolic rate and influencing neighboring cell function [28–30]. For example, short telomeres may affect pancreatic β-cell metabolism directly [7]. In T2DM, studies have demonstrated a reduction in both global β-cell signaling and production of insulin in the setting of pancreatic cell senescence [7]. However, the relationship between LTL and obesity [31] and diabetes [32] has been questioned. The severity of disease complications may also interact with telomere length regulation. For example, in T2DM with complications such as a previous myocardial infarction (MI), telomere length is shorter, compared to the presence of T2DM without MI per se [18].

## Is the interaction between telomere length and T2DM influenced by genetic variation?

Several studies have reported positive associations between LTL and T2DM. LTL has been shown to be shorter in patients with T2DM and also correlates with time of onset and duration of disease [19, 33, 34]. However, a Chinese population-based study found no relationship between LTL and either the time of onset or the duration of T2DM [35]. Mechanisms other than diabetes may be responsible for shorter telomeres in a Chinese population. Genetic regulation of telomeres could potentially explain telomere shortening and an increased risk for T2DM. Current conceptual thinking is that shorter telomeres may predispose to, or accelerate, biological aging-related disease by underpinning a bidirectional cause-and-effect relationship between disease and telomere length [36]. Possible mechanisms that could result in bidirectional changes in both metabolic disease progression and telomere shortening would be functional expression or activity changes in telomere length-related genes and pathways and alterations in cellular oxidative stress.

### Uncoupling protein association

The three common isotypes of UCP are UCP1, mainly expressed in brown adipose tissue, UCP2, expressed widely in most cells and UCP3, expressed mainly in striated muscle [37]. UCP2 has been implicated in diabetes risk and interactions with telomere-pathways [13, 38].

UCPs are located in the inner mitochondrial membrane and mediate proton leakage, to regulate the production of ATP and reduce oxidative stress [37, 39]. The high degree of homology at the nucleotide level and the conservation of the exon/intron boundaries among the key UCP genes (*UCP1*, *UCP2*, *UCP3*) suggests they may have evolved from a common ancestor or are the result of gene duplication events [40]. Peroxisome proliferator-activated receptors (PPARs) are ligand-activated transcription factors involved in the regulation of glucose/lipid metabolism [41] and are pivotal in the control of transcription of the UCP family of genes [42, 43] Interestingly, pre-clinical studies in Otsuka Long-Evans Tokushima fatty (OLETF) rats, that develop T2DM, have shown that PPAR-γ is also effective at elevating telomerase activity and telomere-associated proteins [41], ameliorating the effects of T2DM on telomere shortening.

Two studies have explored the association between the *UCP2* gene variant −866G < A (rs659366), T2DM and telomere length [13, 38]. One study covered a range of ethnic backgrounds [13] (Caucasian, South Asian and Afro-Caribbean) while the other focused on a Han Chinese population [38]. LTL and the −866G < A UCP2 variant were found to be independent risk factors for T2DM [38]. The −866G < A UCP2 variant was also particularly related to shorter LTL in patients [13]. These studies were some of the first to suggest a possible association between the efficiency of mitochondrial UCP regulation of reactive oxygen species [44, 45] and shorter telomere length in T2DM.

### Telomerase and shelterin association

Eukaryotic chromosome stability requires telomere maintenance by the RNA-protein telomerase complex, which is responsible for reverse transcriptase activity for the maintenance of telomere ends, by adding DNA sequence repeats [46]. The telomerase holoenzyme is composed of both protein and RNA, i.e. telomeric reverse transcriptase (TERT), telomeric RNA component (TERC) and other factors, e.g. telomerase protein component 1 (TEP1). The function of telomerase relies on proper expression, maturation, trafficking and assembly of these components [47]. TERT is the catalytic subunit of the enzyme telomerase that catalyses the addition of multiple 6 bp (TTAGGG) telomere repeats, using TERC as a template [48]. TEP1 is a component that facilitates telomerase activity.

Moreover, the shelterin complex is recognized as being important for the protection of telomeres and for the regulation of telomerase activity [49]. Shelterin is comprised of six important components: telomere repeat factor 1 and 2 (TRF1 and TRF2), protection of telomere 1 (POT1), repressor/activator protein 1 (RAP1), TRF1- and TRF2- interacting nuclear protein 2 (TIN2), and tripeptidyl-peptidase 1 (TPP1) [50]. The TEL-patch of amino acids, on the oligonucleotide/oligosaccharide-binding folds of the shelterin component TPP1 is essential to recruit telomerase to telomeres [50]. While important, there is a paucity of basic biological understanding of how the shelterin complex components may interact with mitochondrial function and induce insulin resistance [51, 52], although TIN2 has been shown to be post-translationally processed in mitochondria and regulates mitochondrial oxidative phosphorylation [53].

Zee and associates explored 11 telomere pathway genes and their relationship to development of T2DM in a longitudinal cohort of 22,715 healthy middle-aged Caucasian females, free of diabetes or cardiovascular disease at baseline [54]. Gene pathways explored in this cohort were telomere maintenance genes, all selected on the basis of having had a prior association with LTL. During a follow-up period of 13 years, 1445 participants developed T2DM. Results showed that, using haplotype-block analysis, there is an association between several pre-specified haplotypes of TRF1 and TEP1 with T2DM risk. When SNPs were considered individually, SNPs (four each from TRF1 and TEP1 and one each from TPP1 and TRF2) had differential association with T2DM risk. Only one SNP–TRF2 rs4783704, remained significant after correction for repeated measures. Absolute changes in LTL were unfortunately not determined in this study.

Telomerase specifically recognizes and elongates the ends of telomeres using the RNA molecule template, TERC [55]. TERC is known to be essential for telomere homeostasis [48]. Clinical and epidemiological studies have revealed that mutations in TERC lead to premature telomere shortening and limited TERC activity result in accelerated shortening of telomere [56, 57]. Shen and associates have explored telomere pathway genes in relation to T2DM, in particular two SNPs, rs12696304 and rs16847897, located near TERC at 3q26 [58]. These SNPs were examined in 4016 Shanghai Han Chinese participants; 1936 T2DM patients and 2080 healthy controls. Both of these major alleles in regression models were associated with between 3 years (rs12696304) and 4 years (rs16847897) of advanced age-related telomere attrition adjusted for diabetes status [58]. In summary, these two TERC-related SNPs correlate with LTL of both T2DM patients and controls. Moreover, data from functional studies, where TERC is knocked down or overexpressed using exogenous mutated TERC, suggest non-telomere maintenance functions for TERC, such as cell cycle control and p53-dependant pathway activation [59, 60]. p53 pathways are of interest as they provide a mechanism linking mitochondrial damage, ROS production and DNA damage responses, and related cellular growth arrest phenotypes during cellular senescence [61–63]. Thus, it is not surprising to find that a TERC SNP related to both increased cardiovascular disease and also T2DM is characterized by cellular senescence.

### Genome-wide association studies’ findings

General population studies have identified several genes through genome-wide association studies as a locus for cross-sectional individual variation in LTL [64, 65]. A novel relative telomere length locus at chromosome 16q21, an intronic variant (rs74019828) in the *CSNK2A2* (Casein Kinase 2, Alpha Prime Polypeptide) gene, was found in South Asians [65]. Phosphorylation by CSNK2A2 plays an important role for regulation of telomere length homoeostasis [63]. Oligonucleotide/oligosaccharide-binding folds containing 1 (OBFC1) is involved in the initiation of DNA replication and also functions in a telomere-associated complex [66]. Several GWAS studies have associated OBFC1 with LTL [64, 67]. In the latter population study, an OBFC1 genotype (rs4387287) was found to be associated with both T2DM and LTL, where the mean difference in LTL across the homozygous genotypes approximates 400 bp in men and 180 bp in women [64]. The OBFC1 genotype (rs4387287) is associated with a defined rate of LTL shortening in the general population, circa 20–30 bp/year [68]. OBFC1 is of particular relevance since although it is unrelated to oxidative stress or mitochondrial function, it is directly engaged in telomere biology [64, 69], suggesting that OBFC1 is able to regulate telomere length and/or function [64].

The relevance of OBFC1 is supported by the finding of Maubaret et al. [70], where an OBFC1 haplotype (rs10786775G and rs11591710C) was associated with lower risk of coronary heart disease. They also found a TERC haplotype (rs12696304G, rs10936601T and rs16847897C) was associated with both a lower risk of coronary heart disease and T2DM.

Another independent study also provides positive evidence for the association between OBFC1 and LTL. You et al. examined rs4387287 on the *OBFC1* gene, which is associated with LTL, and its association with T2DM development. In addition, another nine genes, including TERT, TERC, TEP1, TRF1, TRF2, POT1, and TPP1 were exmined [71]. The study population examined was 82,069 ethnically diverse postmenopausal women who had no prior history of chronic diseases (including diabetes or cardiovascular disease), of which over 6 years of follow-up 1675 developed T2DM. Overall, You et al. concluded that LTL was found to be weakly associated with diabetes risk but was not independent from known epidemiological risk factors.

In summary, this review suggests that short LTL has been identified in a limited number of population studies as a risk factor for the development of T2DM. The shortening of LTL relates to both OBFC1 genotypes and telomerase genotypes such as TERC. Interestingly, the interaction between short telomeres and T2DM risk appears to involve mitochondrial dysfunction as an intermediate process, suggesting a mitochondrial axis where underlying gene regulation of telomerase-related pathways is important. We suggest that genetic heterogeneity within UCPs may directly affect oxidative stress that feeds back to influence the fine balance of telomere regulation and cell cycle regulation. This in turn may, in part, be related to both the development of T2DM and enhancing the rate of complications once the disease is present. The interaction of cardiovascular disease in association with the development of T2DM is important and is likely to associate with some genetic components of telomere regulation, but not with all. Overall, the accumulated data in this review illustrates the emerging influence of genetic factors on LTL and T2DM and interplay with mitochondrial function. We suggest that there is a likely need to explore the biological and pathological interactions between UCP, telomerase and shelterin regulatory pathways. These studies are required to adequately describe the precise role of mitochondria on LTL shortening with respect to both acute and chronic pathological events. A greater understanding of the genetic influences on telomere length and T2DM is likely to facilitate risk stratification amongst patients, as genomics is progressively embraced as a clinical tool in the management of this chronic illness.
